# Supplementary material for: Embryo-restricted responses to maternal IL-17A promote neurodevelopmental disorders in mouse offspring
Source: Mol Psychiatry. 2024 Oct 10;30(4):1585–93. doi: 10.1038/s41380-024-02772-6 (PMC11919734; doi:10.1038/s41380-024-02772-6)
Supplement: Supplementary file 1 — Supplementary Figure Legend [file 41380_2024_2772_MOESM1_ESM.docx]

**Supplementary Figure Legends**

**Supplementary Fig.1 Th17 and Treg cells are not impacted in adult female mice prenatally exposed to IL-17A. a)** Gating strategy for identification of intestinal lamina propria and splenic Th17/Treg/RORγt+Treg cells by flow cytometry in female mice. **b)** Representative FACS plots depict RORγt and Foxp3 expression by T cells (gated on: CD11b^-^F4/80^-^TCRβ^+^CD4^+^ cells). Numbers within plots indicate mean±SD frequencies of cells in the adjacent gate. No statistically significant differences were noted in frequencies between groups. **c)** numbers of Th17, Treg and RORγt^+^ Treg cells in the small intestine (top), colon (middle) and spleen (bottom). Graphs show pooled data from two (intestines) and four (spleen) independent experiments. Small intestine: n=8 from 2 litters (white) and 7 from 3 litters (red); Colon: n=9 from 4 litters (white) and 10 from 5 litters (red); Spleen: n=17 per group from 6 litters (white) and 8 litters (red). To normalize data from independent experiments, the cell number of each T cell subset was divided by the number of million cells in the sample. Datapoints represent individual mice. Bar graphs indicate mean±SD. ns=p>0.05 two-tailed unpaired t-test. Control=IL-17RA^KO/WT^ offspring born to IL-17RA^KO/KO^ dams (white). IL-17A imprinted=IL-17RA^KO/WT^ offspring born to IL-17RA^KO/KO^IL-17A^OE/WT^ dams (red).

**Supplementary Fig.2 Extended analysis of the ultrasonic vocalization (USV) profile of IL-17A-imprinted pups.** a) Example USV profile highlighting the assessed parameters for extended analysis. Red dotted boxes mark individual vocalizations. The quantification shown in c)-e) was performed by averaging the duration, bottom frequency (lower red box edge), and peak frequency (upper red box edge) values of all emitted vocalizations per pup. b) Number of vocalizations. Bar graphs represent median±95%CI. ns=p>0.05 two-tailed unpaired Mann-Whitney test. c) USV duration d) USV low frequency. e) USV high frequency. f) USV frequency range, quantified by subtracting the mean USV low frequency from the mean USV high frequency of an individual pup.

b)-f) Data points represent individual pups. Males: n=17 from 6 litters (white) and 17 from 8 litters (red). Females: n=24 from 7 litters (white) and 10 from 8 litters (red). c)-f) Bar graphs indicate mean±SD. *=p<0.05, ns=p>0.05 two-way ANOVA with Šídák's multiple comparisons test. Control=IL-17RA^KO/WT^ offspring born to IL-17RA^KO/KO^ dams (white). IL-17A imprinted=IL-17RA^KO/WT^ offspring born to IL-17RA^KO/KO^IL-17A^OE/WT^ dams (red).

**Supplementary Fig.3 Proposed mechanism by which maternal IL-17A mediates behavioral disturbances in offspring.** IL-17A present in the maternal blood (1) is translocated across the placental barrier (2) which is formed by four embryonic cell layers (Pink=Trophoblast giant cell layer; Green=Syncytiotrophoblast bilayer; Blue=Endothelial cell layer). Maternal-derived IL-17A is distributed within the embryo, leading to prenatal imprinting to IL-17A (3). Exposure to IL-17A in the prenatal period leads to long-lasting behavioral disturbances (4).
