# Supplementary figures and images for: Embryo-restricted responses to maternal IL-17A promote neurodevelopmental disorders in mouse offspring
Source: Mol Psychiatry. 2024 Oct 10;30(4):1585–93. doi: 10.1038/s41380-024-02772-6 (PMC11919734; doi:10.1038/s41380-024-02772-6)

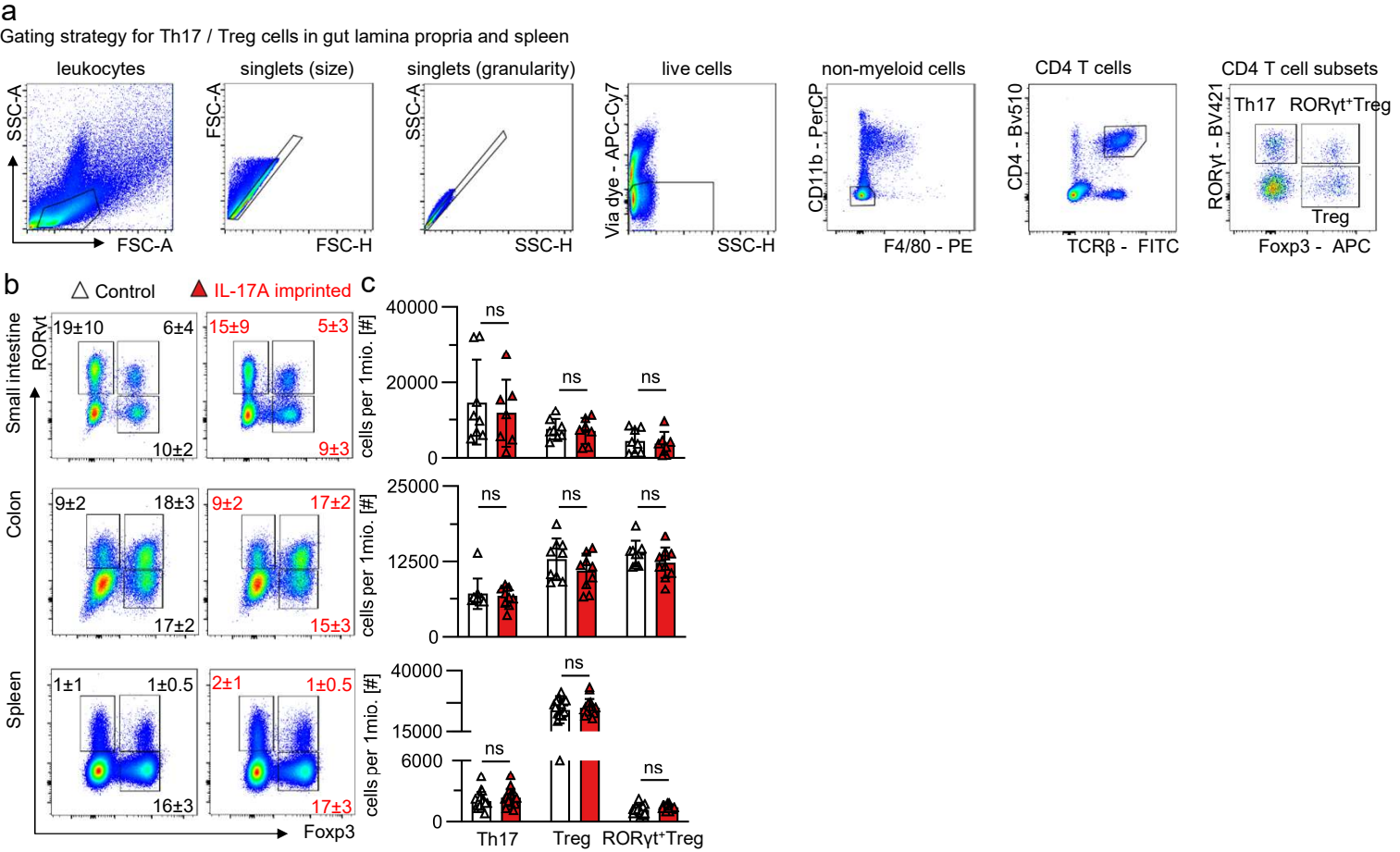

Suppl. Fig.1

Supplement: Supplementary file 2 — Supplementary Figure 1 [file 41380_2024_2772_MOESM2_ESM.pdf]

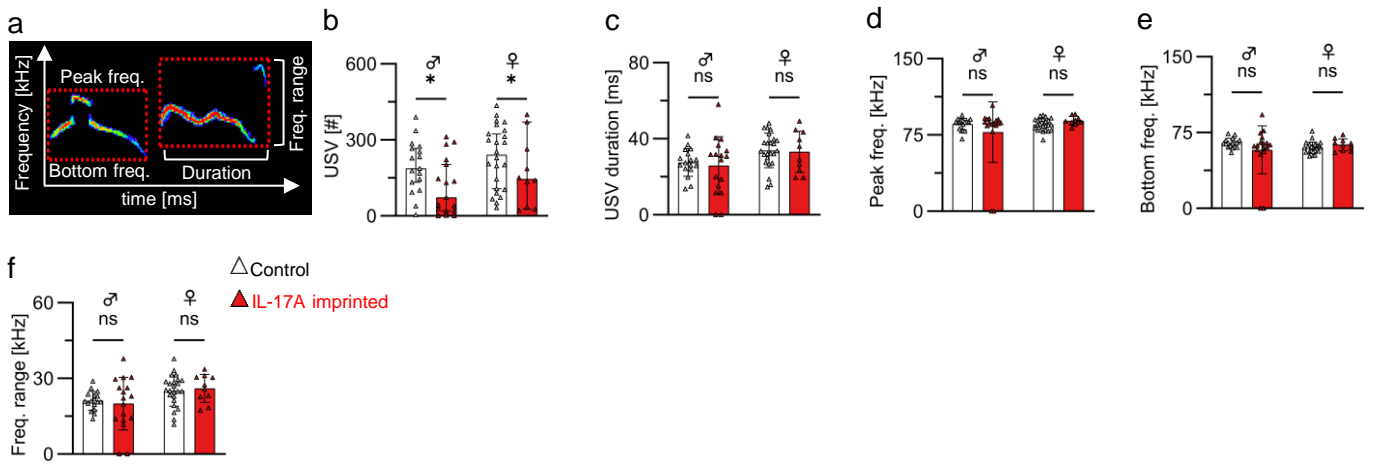

Suppl. Fig.2

Supplement: Supplementary file 3 — Supplementary Figure 2 [file 41380_2024_2772_MOESM3_ESM.pdf]

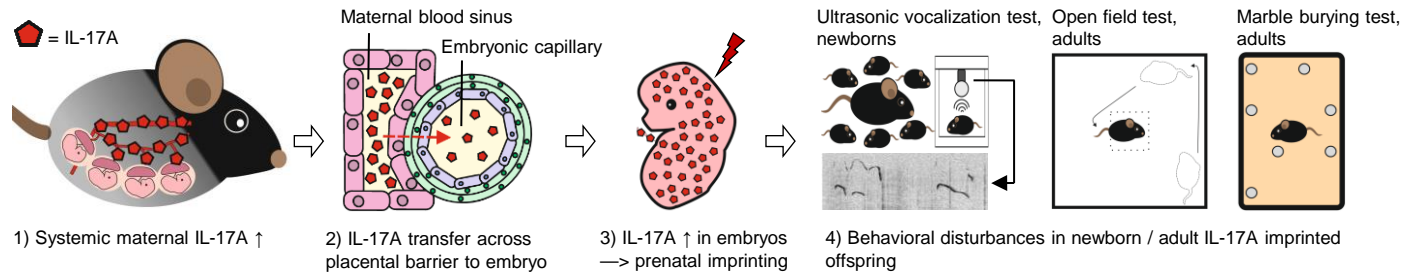

**Suppl. Fig.3**

Supplement: Supplementary file 4 — Supplementary Figure 3 [file 41380_2024_2772_MOESM4_ESM.pdf]
